# Corrosion-driven droplet wetting on iron nanolayers

**DOI:** 10.1038/s41598-023-45547-9

**Published:** 2023-10-25

**Authors:** Aurelien Ricard, Frederic Restagno, Yun Hee Jang, Yves Lansac, Eric Raspaud

**Affiliations:** 1 Laboratoire de Physique des Solides, Université Paris-Saclay, CNRS, 91405 Orsay Cedex, France; 2 GREMAN UMR 7347, CNRS, INSA CVL, Université de Tours, 37200 Tours, France; 3 Department of Energy Science and Engineering, DGIST, Daegu, 42988 Korea

**Keywords:** Chemical engineering, Corrosion

## Abstract

The classical Evans’ drop describes a drop of aqueous salt solution, placed on a bulk metal surface where it displays a corrosion pit that grows over time producing further oxide deposits from the metal dissolution. We focus here on the corrosion-induced droplet spreading using iron nanolayers whose semi-transparency allowed us to monitor both iron corrosion propagation and electrolyte droplet behavior by simple optical means. We thus observed that pits grow under the droplet and merge into a corrosion front. This front reached the triple contact line and drove a non radial spreading, until it propagated outside the immobile droplet. Such chemically-active wetting is only observed in the presence of a conductive substrate that provides strong adhesion of the iron nanofilm to the substrate. By revisiting the classic Evan’s drop experiment on thick iron film, a weaker corrosion-driven droplet spreading is also identified. These results require further investigations, but they clearly open up new perspectives on substrate wetting by corrosion-like electrochemical reactions at the nanometer scale.

## Introduction

Droplets standing on a surface is not only a matter of art but also a matter of fundamental and applied sciences (paintings, inkjet printing and material protection such as corrosion inhibition, among others). Gravity pulls the liquid down from the surface to a level called the capillary length, where interactions between molecules start to prevail^1^. Liquid/solid interactions at the molecular and nanometer levels govern the interfacial properties that control its spreading. There are still many open questions at this level. For example, wettability, structure and dynamics of water at metal interfaces are still under study at a detailed molecular level (see for instance^2^). Interfacial arrangement and orientation of molecules like water but also spatial distribution of dissolved ions are intimately coupled with the metallic surface potential, and such coupling creates additional difficulties in predicting the droplet shape^3^. Numerous electrodynamical couplings can alter with time the distribution and local arrangement of charges in the conductor and of ions in the liquid^4–7^.

A number of studies have demonstrated that spatial gradients in such solid/liquid interactions (hydrophilicity^8^, stiffness^9^ and surface tension^10,11^) can make droplets move over a flat horizontal surface of a non-porous solid material. Surface chemistry and surface texture have also been found to drive a spreading of droplets^12–16^. In particular, the “reactive spreading” observed at high temperature depends on diverse processes such as interfacial reaction, mutual dissolution, species diffusion and fluxing^12–14^. Note that the spreading of micrometer-sized droplets has also been described by tension line effect at a sub-millimeter level^17,18^.

Observing corrosion of a metal surface under a salt-containing aqueous droplet corresponds to the classical “Evans’ drop” experiment^19^. In the classical picture (see Figure S1, Supplementary Information (SI)), corrosion involves an electrochemical reaction where electrons coming from metal oxidation reduce dissolved oxygen molecules. The process is thus sensitive to oxygen gradient (differential aeration); “a drop of brine placed on a steel plate causes corrosion below its center; but near the edge of the drop, where oxygen has best access to the metal, there is no attack at all”^19^. This spatial separation of reactions induces a pH gradient and accelerates the pitting corrosion^20^. Further reactions lead to the formation of iron oxides and oxy-hydroxides, green and brown rust. Both electrochemical reactions at the metal surface and further reactions inside the liquid droplet produce a dynamical system with spatial chemical gradients, potential gradients, diffusion and convection of species together with water movements^21–23^. However, only a little is known about the droplet spreading due to corrosion, e.g., lateral expansion of a NaCl-containing droplet concomitant with pit openings on a Cu surface^24^, formation of micro-droplets and lateral expansion of a thin water layer around the primary droplet in high-humidity conditions^21^, expansion of micro-droplets^25^, and influence of CO_2_ level on the secondary spreading dynamics^26^.

We report here the spreading, at room temperature, of a salt-containing aqueous droplet standing on the macroscopic smooth surface of a 10-nm-thick reactive metallic substrate. Previous studies have reported the stability and uniformity over large areas of such two-dimensional (2D) films^27–30^. Without any chemical or physical defects, an oxide layer would form at the top of the film and protect the underlying pure iron Fe(0) from aqueous corrosion. Here, the work is performed on samples having a defective protective layer, and we show how pits on nanofilms propagate and how this propagation drives the droplet spreading. Additional experiments performed on a bulk iron sheet indicate (dis)similarities in the droplet spreading and corrosion process.

## Material and methods

### Nanofilms fabrication

The substrate surface must be perfectly clean with the weakest possible roughness to transform the deposit into ultrathin and uniform layers of iron over a large area. We cleaned 24 mm × 24 mm microscope glass coverslips (Thermo Scientific) using CaCO_3_ suspended in a surfactant (RBS, 2% volume mixed in water). We rubbed the glass coverslip with cotton that we immersed into the suspension. We performed a second similar cleaning without CaCO3 and one more with Milli-Q (MQ) water (resistivity 18.2 MΩcm). We dried the substrate with a nitrogen gun under a protected atmosphere.

We then deposited 10 nm of iron (99.95% pure iron pellets, Kurt J. Lesker) using a Joule effect evaporator under low pressure conditions (10^–7^ mbar). In order to promote iron adhesion to the substrate, 5 nm of titanium were deposited before iron (99,99% Ti pellets, Neyco) directly onto the glass coverslip. We controlled metal deposition thickness with a quartz balance integrated into the evaporator. Samples were then exposed to ambient air and stored for at least two weeks, the time needed to form a protective oxide layer in the case where no defects are present^28–30^.

After this period, a few samples were inspected by optical microscopy. As shown in Figure S2, Supplementary Information (SI), we noticed the presence of a small number of microscopic holes within the iron layer together with isolated and sparse contaminations standing over the surface.

### Salt solution and water preparation

We weighted 3.78 g of solid KCl salt (SIGMA, 99%) using a precision scale, and dissolved it into a beaker with a few milliliters of MQ water. We transferred the solution into a 200 ml volumetric flask, and completed the volume with MQ water (performing one agitation when 75% of the volume was completed). We stored this 250 mM KCl solution in 50 ml Falcon tubes at room temperature.

### Contact angle measurement

We performed static and dynamic contact angle measurements on Ti-5 nm, Fe-10 nm and Ti-5 nm-Fe-10 nm samples, using a commercial setup (Krüss, Drop Shape Analyzer DSA30) and its associated software (Advance DSA4). Only droplets containing 250 mM KCl were studied. Experiments were performed just after their deposition to record the initial states.

In the static mode, a syringe was used to deposit a 5 µl droplet onto the nanofilm. After imaging the droplet, the software fitted the hemisphere profile and measured two contact angles (for each side of the droplet, see Fig. S3A, SI). We further define the static contact angle by the one measured just after water droplet deposition (see Table S1, SI).

In the dynamic mode, we measured the advancing contact angle by increasing the droplet’s volume from 10 to 30 µl and the receding contact angle by reducing the droplet’s volume from 30 to 10 µl within 20 s (see Fig. S3B, SI). Only the final angles reached at 30 µl (advancing) and 10 µl (receding) after a few minutes are reported in Table S1, SI.

### Corrosion experiment

We performed iron nanofilms corrosion experiments in a homemade humidity chamber, made by sticking Plexiglas (polymethylmetacrylate) walls and lid together with tetrachloromethane (dimensions 10 cm × 10 cm base and 2 cm height). The chamber was set on a plexiglass base with a rubber seal to close it. High humidity (> 95%) permitted to reduce the water evaporation from the droplet. The room temperature was set to 21 °C. One hour before the experiment, we placed several water tanks to saturate humidity, and closed the chamber with the iron nanofilm inside. Relative humidity was monitored during the first experiments by an incorporated sensor (Sensirion SHT25). We then deposited a 70 µl KCl droplet with a needle through a little opening (3 mm diameter) and imaged corrosion evolution with 3 cameras (Basler acA2440-75uc, Basler acA3800-14um, IDS U3-3580LE-C-HQ) as shown in Figure S4, SI. The camera placed below the sample imaged the corroded area by light transmission (bottom view) while the two others (side and oblique), located above the nanofilm, imaged the droplet profile. A LabVIEW program took a picture with each camera at a chosen rate (usually every 120 s). Note that 13 preliminary experiments (not shown), during which we optimized the experimental protocol and conditions, revealed the phenomena reported here.

The last set of experiments, reported here, was performed using a 0.1-mm-thick bulk iron sheet (purity Fe 99.5%, Goodfellow). We proceeded using the same chamber and the same protocol as described above.

### Image analysis

Due to inhomogeneous lighting conditions and some specificities of our experiment (multiple edges), we developed our own python code based on classical principles of edge detection and which includes personalized analysis as compared with classic algorithms. It allowed us to analyze experimental movies by tracking the growth of corrosion pits’ borders. We converted each picture into binary format and defined the corrosion front as the interface between 0 and 1 pixels using a Laplace operator based Python function^31^ defining the front line to track between each image. To avoid redundancy, we smoothed it and took into account mass centers far enough from one another.

To do so, each pixel was replaced by the mass center of the line’s pixels within a disc of a radius *R* = 2.3 pixels. We performed every velocity calculation on a mass center, and all mass centers closer than *R* to the former one were ignored in further loops.

At each time *t* and for each mass center of coordinates *R*(*t*), we calculated the local tangent to the front line by linearly adjusting coordinates of its five nearest neighbors, thus obtaining the local normal to the front line. Knowing the perpendicular to the local tangent line, we then detected, on the normal line, coordinates of the nearest mass center we could find in the corrosion front detected in the image pictured 18 min later (also considering mass centers). We then defined local front velocity by the ratio between distances that separated the two mass centers. This way, we defined local corrosion front velocities at each time.

We could next sketch the speed histogram at each time, define a mean speed as the histogram Gaussian fit mean, and follow its evolution with respect to time. We defined error as Gaussian standard deviation.

## Results and discussions

**Figure 1:**
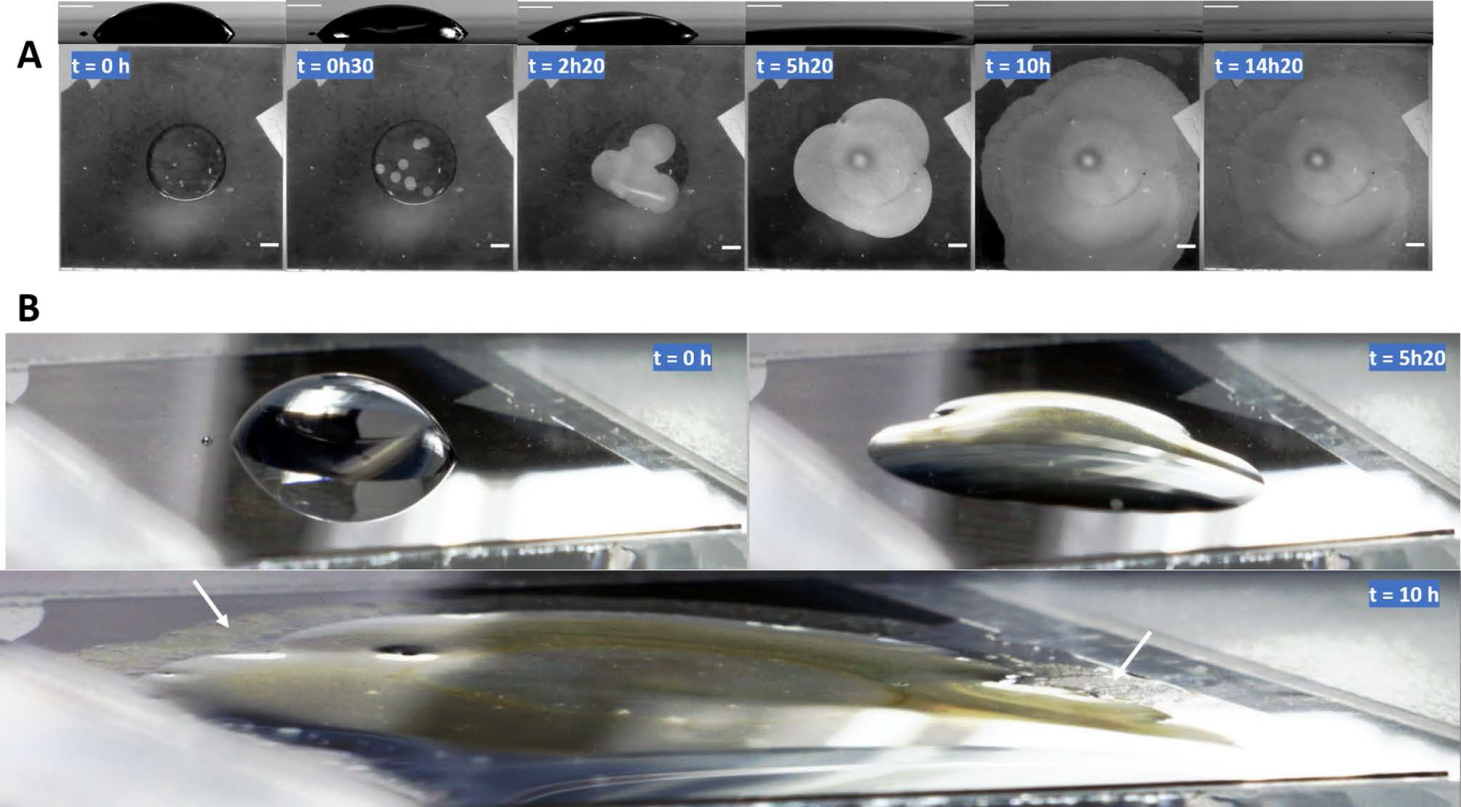
Series of pictures showing the time evolution of the system. (**A**) Side view (upper picture) of the droplet profile and bottom view (lower picture) of the nanofilm degradation (a bright pixel meaning corroded area) at six different times. Scale bars: 2 mm. Note: a bright area surrounded by a dark circle is visible in the center of the last three pictures (bottom views). It corresponds to the image of the Plexiglas chamber opening used for droplet deposition with a syringe (see Fig. S4). (**B**) Oblique images showing the droplet spreading at three different times. White arrows show corroded areas located outside the droplet.

As reported in a previous work^30^, the optical semi-transparency of nanofilms allowed us to simply monitor the corrosion process by optical means (see Material and Methods). Figure 1 presents time evolution snapshots of the corrosion and wetting processes, with side and bottom views in Fig. 1A and oblique view in Fig. 1B; note that the image contrast has been increased and that the two first oblique views have been distorted for clarity reasons. Movie S1– Supplementary file shows the complete time-lapse sequence.

At *t* = 0 h, left pictures, we deposited a droplet of 250 mM KCl on a nanofilm Ti–Fe of 15 nm in thickness, composed by 10 nm of Fe deposited on a pre-evaporated 5 nm Ti adhesion layer (read “Material and methods” for details). Adjusting tangents to the droplet profile near the substrate indicates initial contact angles very close to 76° and 69° corresponding to the static and advancing contact angles, respectively, that were measured using a commercial contact angle meter (see “Material and methods” and Fig. S3A, B—Supplementary file). The circular droplet shape may be seen from the bottom view. Few bright dots, scattered on the nanofilm surface and mostly located on the area covered by the droplet are clearly visible.

At *t* = 0 h 30, the side view shows small changes in droplet dimensions with a decrease in height and an increase in diameter. The side profile being still symmetric, we interpreted these changes as long-term droplet relaxation^32^ following its deposition combined with evaporation despite high relative humidity. The droplet circular shape is also visible on the bottom view, together with large bright disc-shaped areas. By comparing the bottom views recorded at *t* = 0 h and at *t* = 0 h 30, it becomes evident from the colocalization that bright areas result from the time-evolution of the initial bright dots. As brightness indicates high light transmission resulting from nanofilm degradation, the bright dots correspond to the pits as defined in the corrosion domain. We noticed that the number of pits per droplet depended on how clean was the glass substrate and how uniform was the nanofilm. We observed by optical microscopy that some pits were centered on defects (see Fig. S5); we suspected these defects to be related to the initial defects already present on nanofilms before the experiments (see “Material and methods”).

Between *t* = 0 h 30 and 2 h 20, some side views recorded in color (Fig. S6) indicated droplet’s yellowishness while bottom views show that pits merged and continued to grow. The triple or contact line started to follow the pit corrosion front or the iron dissolution front, as observed on side view at *t* = 2 h 20 (on the left side of the droplet). The deformation process becomes even clearer at *t* = 5 h 20 in Fig. 1A where the droplet flattens due to lateral spreading. The two oblique views shown in Fig. 1B at *t* = 0 h and *t* = 5 h 20 highlight the shape transition from hemispherical to puddle-shaped. Droplet deformation became localized where the corrosion front pined the contact line, with contact angles very different from those measured on the iron nanolayer before corrosion. At that intermediate step, the front drove progressively the spreading of the aqueous droplet. Finally, the last pictures in Fig. 1A, B, recorded at *t* = 10 h and 14 h 20, show that the dissolution front continued to extend while the droplet had reached a final size. White arrows on Fig. 1B point to transparent areas where nanofilm dissolved though being located outside the droplet suggesting the presence of a thin water layer, invisible on pictures, and most likely related to the high humidity level in the chamber. It is similar to the thin water layer surrounding the primary droplet deposited on stainless-steel postulated by Tsuru et al.^21^. Note also the apparent surface irregularities, almost due to the presence of deposits. At the end of the process, the whole nanofilm disappeared.

Experiments carried on four different nanofilm samples exhibited similar features, but with successive steps occurring at times that varied from sample to sample. Similar observations were made when replacing KCl by NaCl salt at the same concentration (not shown). Quantitative data were obtained by processing bottom view images, analyzing gray level values of each pixel. We focused first on the corrosion process across the nanofilm thickness. Previous study already reported corrosion rate values of nanofilms where vertical corrosion was found progressive and uniform^30^. Here, the corrosion front was found as sharp as the nanofilm edge located at the glass coverslip side (see Fig. S7), indicating a transverse corrosion too fast to be measurable in our experimental condition.

**Figure 2:**
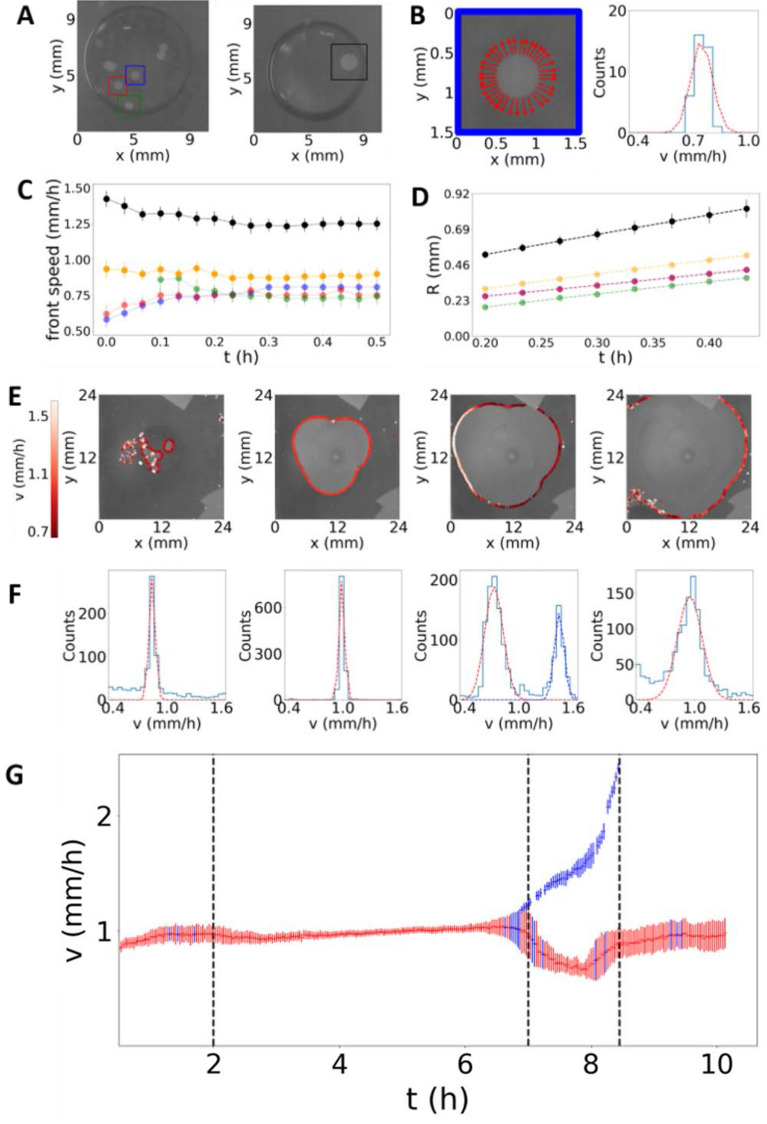
Corrosion evolution through the different steps, (**A–D**) the first step and (**E–G**) the next four steps. (**A**) Images showing the four different pits (from two different experiments) studied in (**B–D**). (**B**) Illustration of the calculated front velocities (left), arrows show the direction of the corrosion front and the relative speed intensity. Corresponding speed histogram (right, blue) with its Gaussian fit (orange dashed line) determining the experimental mean velocity. (**C**) Temporal evolution of the mean front speed extracted for each front (an orange line has been added from a third experiment). Error bars are given by the standard deviation of the fit. (**D**) Calculated radii from corroded areas. (**E**) Velocity distribution along the corrosion front at four different times (from left to right: 1 h, 5h20, 8 h, 10h30) corresponding to the next four successive steps. Note the detection noise in the first (left) and last (right) pictures. (**F**) Velocities histograms at the same four times than in (**E**), and gaussian fit for each peak (dashed lines). Note the very narrow speed distributions during the three first steps (histogram in (**B**) and the two first histograms on the left side in (**F**)) compared to the wide distributions detected during the two final steps (the two last histograms on the right side in (**F**)). (**G**) Temporal evolution of the mean velocity calculated from the Gaussian fit of each peak. When two peaks are detected, two velocities are sketched (red and blue). Vertical dashed lines illustrate the four different corrosion steps following the initial step (**A**–**D**) where pits keep isolated and separated from each other.

Five successive steps (Fig. 2) were identified: when isolated pits (1) grow and (2) merge inside the droplet, (3) when the corrosion front pins the contact line and induces the droplet spreading, (4) when the droplet stops spreading at some locations and continues at others and, (5) at the end of the process when the corrosion front continues to propagate while the droplet stopped spreading.

During the first 30 min, isolated pits start to expand laterally inside the droplet (step 1). Figure 2A–D show the analyses performed on three nearby isolated pits appearing on the same sample (framed with different colors (red, blue and green) in Fig. 2A) and on a single pit that appeared on another sample (black frame). Figure 2B displays the front line velocity vectors (colored in red) superimposed on the pit image (in blue-frame magnification) and the corresponding distribution of speed values. A mean front speed was extracted by fitting the data to a Gaussian distribution (orange dashed-line). The distribution is very narrow, suggesting a uniform front propagation, which is consistent with the circular shape of the different pits. As reported in Fig. 2C, the mean front speeds of the three nearby pits reach similar values that remain constant overtime. Note the absence of mutual effects as the nearby opening pits get closer from each other with time, indicating a propagation process mostly driven by a local phenomenon, *i.e*. the chemical reactions associated with the dissolution process. Analyses performed on different nanofilms lead to the same value for the front speed, of the order of 1 mm/h. As pits open circularly, radius *R* of each pit was also determined by monitoring the corroded-surface area. As shown in Fig. 2D, radii of different pits increase linearly with time, as expected for an opening process occurring at a constant speed.

Figure 2E–G shows the results obtained over the next four successive steps (steps 2–5). To map the corrosion front propagation, Fig. 2E shows velocity vectors, whose magnitude expressed by a gradual color level superimposed on the images recorded overtime. As performed previously (Fig. 2B), the speed distribution was fitted by a Gaussian distribution to extract the mean front speed across the different steps. Results are summarized in Fig. 2F as a function of time. A unique front speed value (of the order of 1 mm/h) is detected during steps 2–3, similar to the value measured for isolated pits opening (step 1). Therefore, pinning of the contact line and droplet spreading do not modify the front speed, the local chemical reactions proceeding at the same rate. However, during the next step (step 4), speed analyses exhibit non-uniformity. The histogram clearly indicates the presence of two characteristic speeds, as illustrated in Fig. 2F, one being lower than 1 mm/h and the other higher than 1 mm/h. Detailed inspection of the speed spatial distribution (Fig. 2E) highlights two regions: (1) Where the front still pined the contact line (close to the nanofilm-glass coverslip edge), the corrosion front accelerated. (2) Where the corrosion front continued to progress without any droplet motion, a small deceleration was observed. Note that the averaged speed over the whole contour front remains equal to 1 mm/h at each instant. As the front speed must be related to the local electrochemical reactions that lead to iron dissolution, in particular the two oxidation and reduction reactions, we can assume a spatial nonuniformity of the two reaction rates, the two reactions being coupled on average. At the end of the process (step 5), the droplet remains immobile, and the corrosion front propagates until the nanofilm edges, again surprisingly at a value similar to the 1 mm/h initial value. This similitude in terms of front propagation would indicate similar chemical and dissolved oxygen composition of the suspected water thin layer and of the droplet. Similar trends were reproduced in other experiments. Duration of each step was different from a sample to another, depending for example on the number of pits and their locations. The last two steps were not observed for one sample (orange-filled symbols in Fig. 2C, D), the third step during which the corrosion front pins the droplet contact line extending up to the end of the process. Iron nanofilm was completely dissolved everywhere, and the droplet fully wetted the whole surface of the sample. On the third sample (black-filled symbols in Fig. 2C, D), we noticed the presence of a very large distribution of speeds during the last steps 4–5 with the presence of three peaks in the speed distribution in step 4.

**Figure 3:**
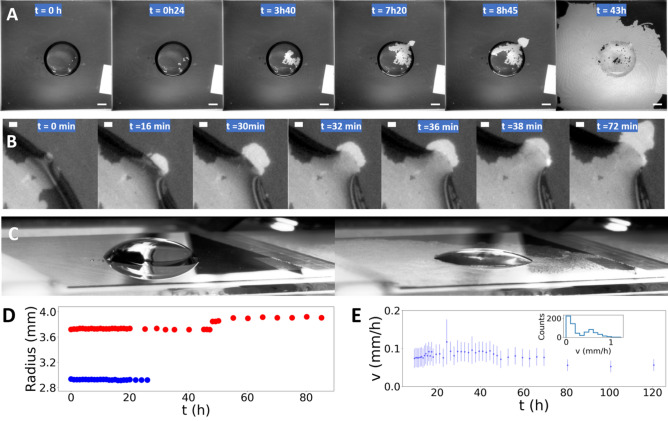
Time evolution of the system when no titanium is present under the iron nanolayer. (**A**) Bottom views of the nanofilm degradation (a bright pixel meaning corroded area) at six different moments. Scale bars : 2 mm. (**B**) Zoomed bottom views showing time evolution of the triple line during iron degradation without titanium. Scale bars : 200 µm, the first picture noted *t* = *0 *min had been recorded at a time *t* = *5.4 *h after the droplet deposition. Other time values noted in the next views refer to the time interval relative to this first picture. (**C**) Oblique images (corresponding to the first and last bottom views) showing the steady droplet during this process. Evaporation effect was noticeable since the experiment lasted a few days. (**D**) Temporal evolution of the radius for a droplet deposited on iron without titanium (red) and for a droplet deposited on titanium without iron (blue). (**E**) Temporal evolution of the front velocity when propagating outside the droplet; only one point out of twenty was preserved for clarity. The inner histogram represents the non-zero speed distribution as detected by the sequence’s analysis at 42 h, indicating a large distribution of speed over 0.5 mm/h during the whole experiment. The most frequent speed value remains equal to 0.07 mm/h as detected at any time, up to 120 h (5 days).

Next, we studied samples where 10 nm iron nanofilms were directly evaporated on the glass substrate, without titanium inbetween. Titanium is used to facilitate iron adhesion as commonly reported in the literature^33^ but it also constitutes an additional conductive layer that can assist the redox reactions. Note however that we did not notice any bias in our previous study performed on nanofilms^30^. Figure 3A shows the typical sequence of images (bottom views) that were recorded over periods that could extend to about one week; Movie S2 corresponds to the complete time-lapse sequence of pictures. Interestingly, wetting and corrosion were found severely impacted by the absence of the titanium layer, although pits started to appear similarly at the very early stage in each type of experiment. As shown at *t* = 0 h, some bright dots spatially distributed over the whole surface covered by the droplet instantaneously appeared. Only some of them expanded with time, as illustrated by the view taken at *t* = 0 h 24. These growing pits were no more circular and presented irregular shapes, indicating different propagation speeds along the peripheral front line. Image inspection reveals that only some parts of the front continued to propagate while other parts were at rest. It resulted in the propagation of irregular and rather elongated shape of corroded areas, with a typical transverse size ranging between 200 µm and 1 mm. Results of a detailed image analysis of the gray level intensity of a pixel belonging to the propagating front are plotted in Figure S8(A & B); the nanofilm disappeared locally at the pixel level within 20 s without titanium, while this period extended over 12 min in the presence of 5 nm titanium. Therefore, the adhesive Ti layer slowed down the propagation process by a factor of 30, which remains puzzling since the propagation should be only sensitive to the chemical reactions involving iron. Even more puzzling is the non-uniformity of the propagation in absence of titanium. Therefore, the titanium layer plays a major role in the corrosion front propagation, a role that might not be simply reduced to the adhesion. On one side, we can hypothesize that the titanium conductive layer facilitates electron exchanges during the chemical reactions. This allows the whole front line to grow and explains why corrosion propagation stops when the conducting layer is constituted of 10 nm of iron only (in fact, even less than 10 nm due to the formation of a 3 nm oxide overlayer^28,29^). On the other side, we can postulate that the absence of a titanium layer decreases iron adhesion to the glass substrate. Water molecules might facilitate the layer delamination from the substrate, promoting the iron layer dissolution as compared with the experiments performed in the presence of titanium. In this case, the leading mechanism would not be considered strictly as a corrosion process of the nanofilm itself, but rather as a delamination process followed by an iron dissolution of the delaminated areas.

Then, as seen in Fig. 3A, t = 03 h 40, some growing pits merge, leaving small islands of remaining iron nanofilm dispersed on the glass surface, and they reach the droplet contact line. An additional time-lapse sequence of magnified views is shown in Fig. 3B, starting from *t* = 5 h 40 when the corrosion front line reaches the droplet contact line. The magnification also highlights irregularities along the front profile. The next two views show the corrosion front propagation without any change in the droplet contact line until the third view recorded two minutes later (32 min), when the local contact line moved suddenly in the direction of the corrosion front. The contact line is pinned by the lateral arrested borders of the propagating area but without fully covering the whole corroded area. A similar process may be observed 4 and 6 min later in Fig. 3B. The motion of the contact line is clearly reminiscent of what Joanny and de Gennes called a strong pinning^34^ when the contact line strongly anchors on a local defect (here, the corrosion front border). The surface energy due to the defect is lower than the elastic energy stored in the deformation of the contact or triple line. The line can be distorted until an instability leads to its progressive smoothening, a dynamical process studied by Ondarcuhu and Veyssié^35^.

Oblique views of the initial and final states (Fig. 3C) illustrate the global non-spreading of the droplet, which is confirmed by the plot in Fig. 3D. Quantitative analyses of the side images allow the droplet radii to be extracted; a plot versus time indicates a constant radius value. Only the droplet height changed with time (Fig. 3C) due to a slow and progressive evaporation over the long period of acquisition (5 days). The droplet spreading over titanium and non-spreading over glass might appear quite puzzling as, paradoxically, we would rather expect the opposite result: water spreads out over clean glass (non-measurable contact angle) rather than clean titanium (static contact angle = 72°, Table S1). But as shown in Fig. S9 by additional drop manipulation on the corroded area, surface hydrophilicity has changed before and after the corrosion process.

Returning to the last image, on the right hand side in Fig. 3A, recorded for this sample 43 h after the droplet deposition, we noticed that the dissolution front continued to propagate over the whole nanofilm. Here again, the absence of titanium profoundly slows down the global front propagation. We also noticed that the front propagation was not uniform, some parts of the corrosion front being completely arrested while others kept moving away from the droplet and also in the orthoradial direction. The stop-and-go propagation was clear for one sample, as illustrated by Figure S8, where the corroded-surface area is plotted versus time. Steps are clearly visible between 40 and 80 h. Finally, we measured the front speed during the overall process (Fig. 3E). Histograms of the non-zero values were extracted and revealed a most frequent speed value that remained quite constant over the whole period and equal to 0.07 ± 0.02 mm/h, a value to be compared with the 1 mm/h front speed value measured in the presence of titanium. The speed distribution was mostly dispersed around this value, except during intermediate periods where histograms present at least two maxima, as shown in the insert of Fig. 3E. In addition, during a long period of time, we noticed on a few samples the presence of growing pits having started at the edge of nanofilm and glass-slide, i.e., far from the droplets, suggesting the presence of a thin water layer.

**Figure 4:**
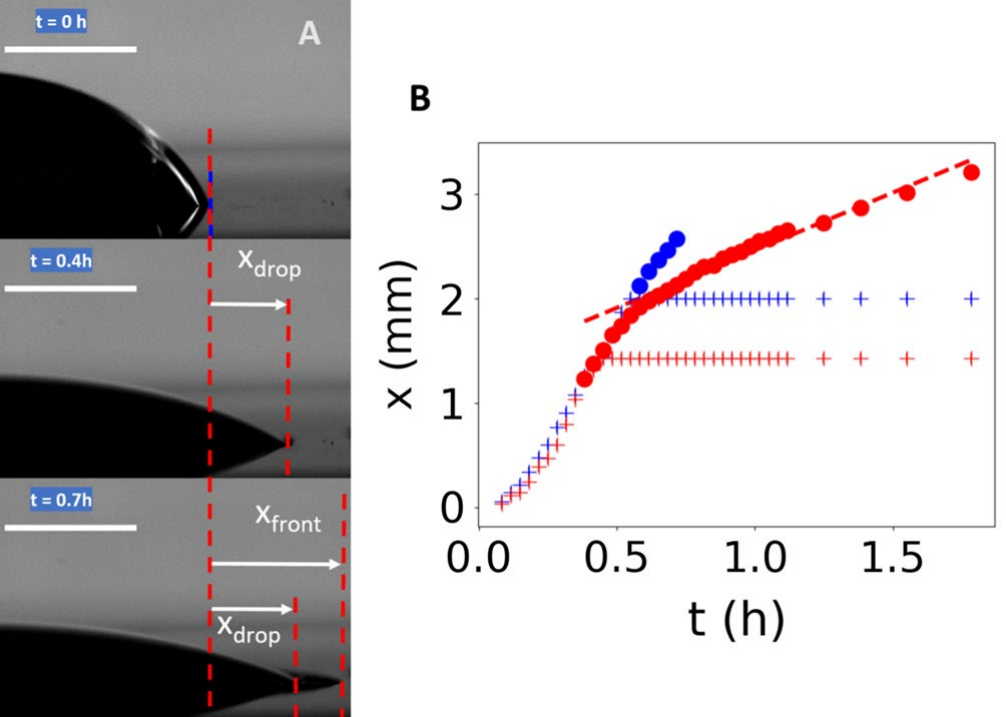
Droplet spreading during corrosion of bulk iron sheet. (**A**) Picture showing the right hand side triple line at three different times. We define a spatial deviation *x* from the initial position on the left and on the right sides of the droplet, and follow the temporal evolution, for both triple contact lines (*X*_*drop*_, second and third pictures) and the corrosion front (*X*_*front*_, third picture). Scale bars = 2 mm. (**B**) Temporal evolution of the spreading (cross symbols) on each side of the droplet (left: blue, right: red) and the propagation of the corrosion front (filled circle symbols) when expanding outside the droplet, noted *x* in both cases. The red dashed line indicates a uniform displacement at 1.1 mm/h.

The last set of experiments were the most classical ones, performed on a 0.1-mm-thick bulk iron sheet (25 × 25 mm^2^). Lateral and oblique views were recorded in the time-lapse imaging assay (Movie S3). We first noticed the short duration of the experiments, less than 10 h, indicating kinetics rather close to those observed on iron nanofilm with an underlying titanium layer. The droplet started to spread immediately after its deposition and kept an apparent circular contact line during its spreading. As shown in Fig. 4, we analyzed in detail the droplet spreading in the side-view pictures. *X*_*drop*_ denotes the lateral position of the contact line relative to its initial position on the left (blue cross) and right (red cross) sides of the droplet. Spreading occurred at the same high speed on both sides (about 4 mm/h) and then stopped after about 30 min, keeping a circular contact line. Another interesting feature is a thin superficial propagating front with the formation of peripheral micro-droplets, in agreement with the previous observations of Tsuru et al.^21,25^. Tang et al*.* suggested that the initiation and propagation of these micro-droplets could influence the corrosion process in itself^25^. Side views allowed us to monitor the front position *X*_*front*_ versus time on both sides of the droplet. Results (filled circles) are plotted in Fig. 4B.. While just emerging from the droplet contour, the front positions continued to vary with the same speed before slowing down to a constant speed, about 1.1 mm/h. Interestingly, this value is equal to the front speed detected on nanofilm experiments, suggesting that the front on bulk sheet could correspond to a propagating front of corrosion on a very thin iron layer over the bulk iron. Note that only local spreading occurred on nanofilm due to expansion of localized pits, which emerged from defects, and gave rise to droplets of different shapes according to the spatial distribution of pits. Here, on bulk iron sheet, quasi-circular shape of the spreading droplet might rather be correlated with the presence of a multitude of defects as expected for iron materials whose oxide layer is not compact enough to protect the underlying metal from corrosion. Nanofilm oxide layer resembles the protective oxide layer formed on steel rather than on pure iron metal.^30^.

In movie S3, during the propagation, we can clearly see the pile of oxide deposits, located at about 1 mm from the contact line inside the droplet and which generates an additional height over which the droplet started to anchor during its evaporation, leading to spatial undulations most probably due to mechanical instabilities. The oxide pile served as structural support during the droplet evaporation and collapse, resembling a volcano shape with a large caldera.

Therefore, spreading differs in the three experimental cases reported here. Theoretically, at thermodynamic equilibrium, spreading at a millimeter level is classically described by a gain in surface energy, the surface tension of the invaded area being lower after the invasion than before. With so many factors influencing the surface nature in itself and its surface tension, it seems quite impossible without any further very detailed analyses to interpret theoretically our experimental results. Among other factors, we can mention:—the thin water layer suspected to cover the non-spreading area, analog of moist. As recalled by de Gennes^1^, its presence over oxides changes drastically the surface tension; —the nature of the corroded area; metal is not stable in contact with water and oxides might regenerate with time; —deposits cover the invaded surface, completely changing its chemical nature; crystallization at the triple line can also lead to a local spreading^36^; —spatial non-uniformities that might also evolve with time. On the liquid drop side, evaporation is known to generate liquid convection and to influence the corrosion phenomenon^37^. The progressive electrochemical reactions themselves might also be considered as an active term. They sustain chemical gradients and fluxes capable of generating liquid flow (see for instance^38,39^), the standing or sessile droplet being itself a dynamical system with internal flows. In this context, it is quite surprising to measure a constant front speed when the corrosion front pins the contact line.

## Conclusion

Corrosion on two-dimensional iron nanofilms induced wetting of salt-contained aqueous solution, provided that nanofilms were deposited on an adhesive and conductive titanium layer. Though the role played by this layer is not clearly identified, this chemically-active process opens up new perspectives on substrate wetting based on electrochemical reactions at the nanometer scale. These observations also bring new elements to our understanding of the Evans’ drop experiment on bulk metal. Results suggest that droplet spreading is intimately related to the superficial two-dimensional corrosion while the formation of microdroplets is due to vertical corrosion across the material thickness, probably related to the amount of oxide deposits and to the pile formation very close to the contact line.

### Supplementary Information

Supplementary Information.

Supplementary Video 1.

Supplementary Video 2.

Supplementary Video 3.
