# Supplementary material for: Corrosion-driven droplet wetting on iron nanolayers
Source: Sci Rep. 2023 Oct 25;13:18288. doi: 10.1038/s41598-023-45547-9 (PMC10600194; doi:10.1038/s41598-023-45547-9)
Supplement: Supplementary file 1 — Supplementary Information. [file 41598_2023_45547_MOESM1_ESM.pdf]

Supporting Information

Corrosion-driven droplet wetting on iron nanolayers

Aurelien Ricard, Frederic Restagno, Yun Hee Jang, Yves Lansac and Eric Raspaud*

Contents :

Figures S1-S9

Table S1

Movies S1-S3, legends

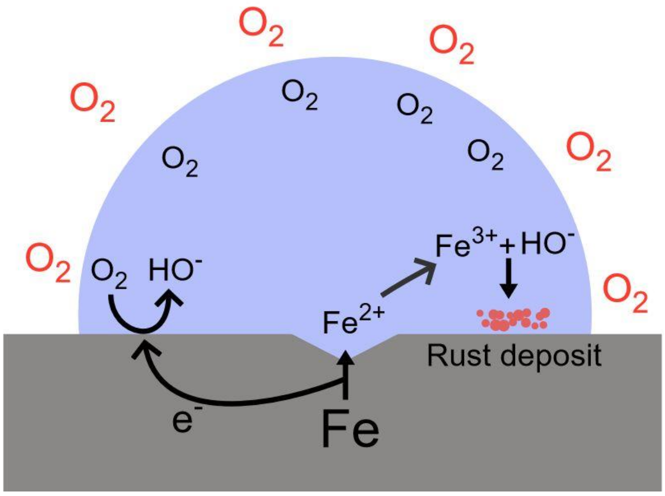

Figure S1 : Schematic illustration of the classic corrosion Evans' drop created with chemix.org. An aqueous droplet is placed on bulk iron. Owing to differential aeration (oxygen concentration is higher at the periphery than at the center of the droplet), anodic and cathodic reactions occur at different locations. One one hand, oxygen reduction reaction occurs at the periphery, where access to oxygen is facilitated, and produces hydroxide ions. On the other hand, iron is oxidized at the droplet center, where the pH decreases, and becomes Iron (II). Then Iron (II) diffuses towards the periphery of the droplet, and can be oxidized again by dissolved oxygen. When iron ions and hydroxide ions meet, rust is formed and precipitates, creating a characteristic ring around the droplet center.

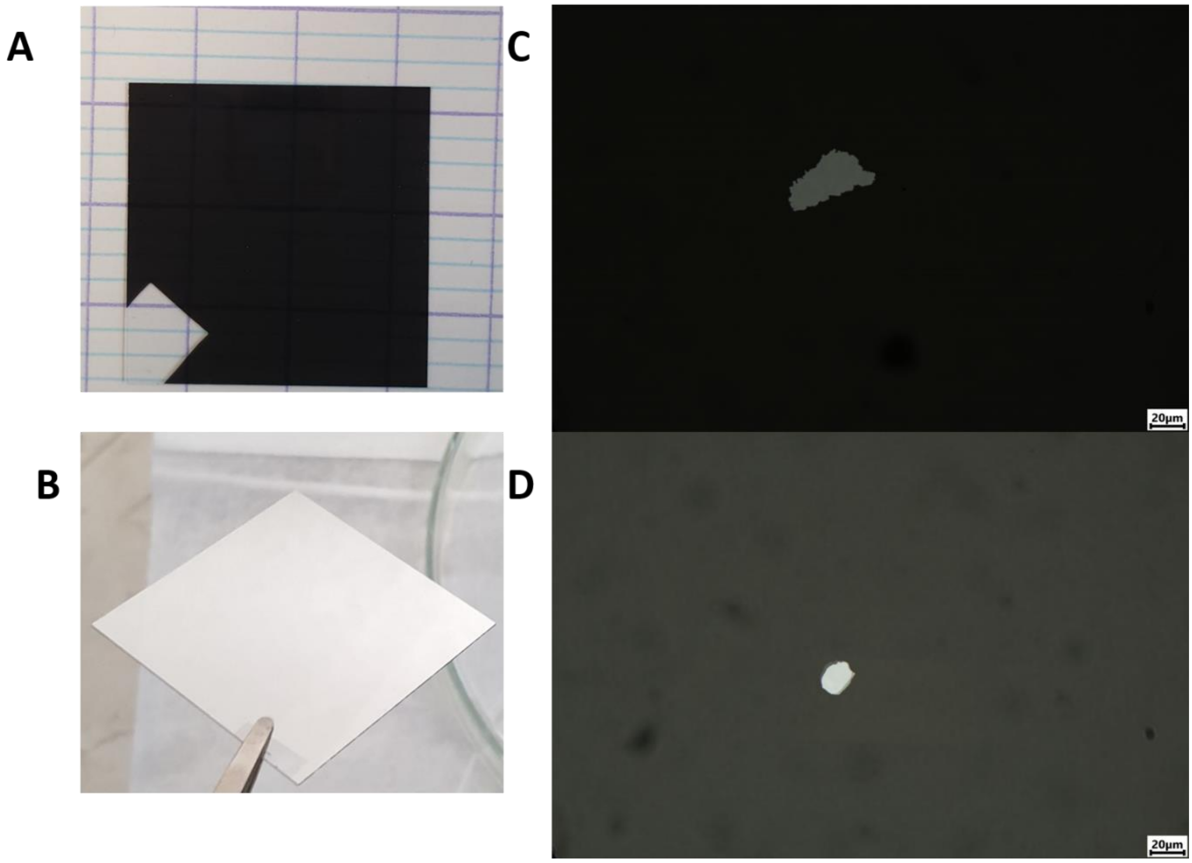

Figure S2 : Pictures of Fe/Ti (10nm/5nm) nanofilms. **A.** Macroscopic picture showing a homogeneous and semi transparent 24mmx24mmx15nm nanofilm. **B.** Macroscopic picture showing the reflexion of light by a 24mmx24mmx15nm nanofilm. **C.** Optical microscopy image by transmission showing an initial defect. A bright area is equivalent to an absence of nanofilm. **D.** Same as **C** for another defect on the same nanofilm.

A

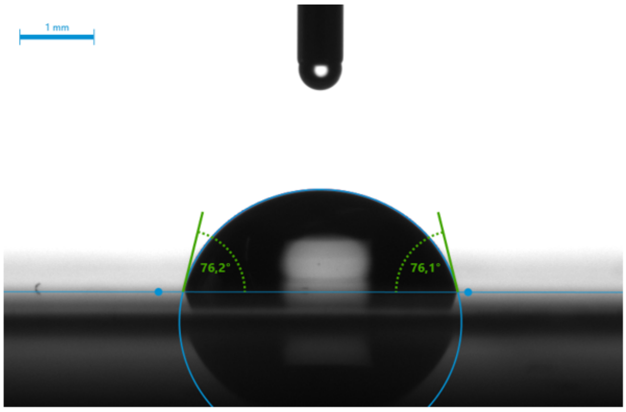

B

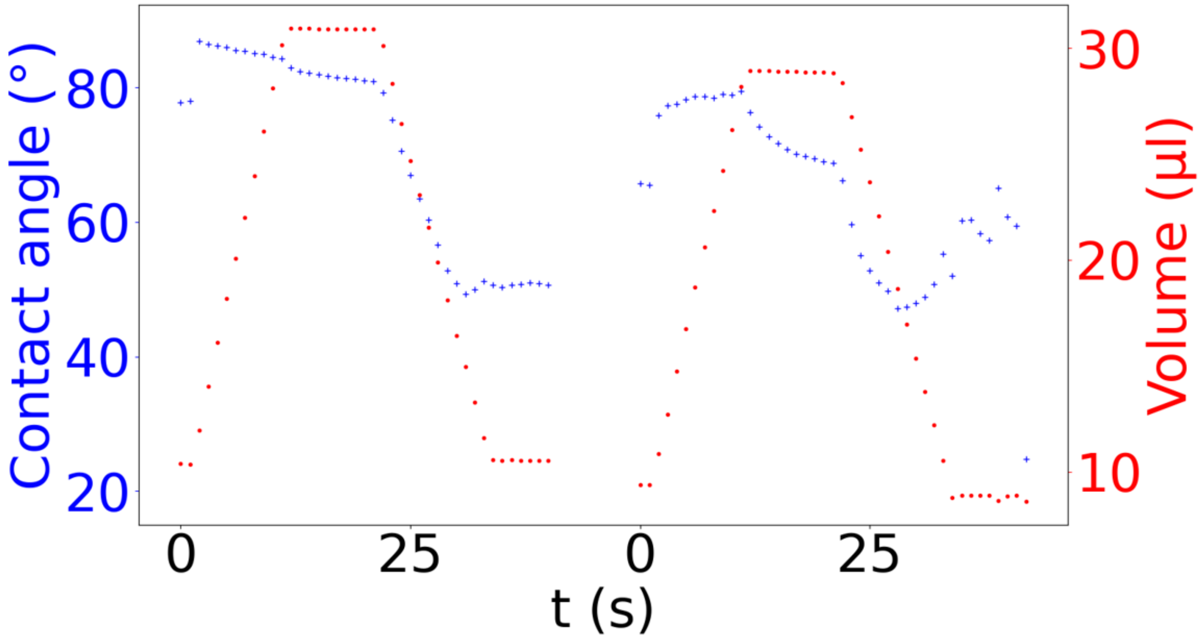

Figure S3 : Dynamic contact angle measurement. **A.** Contact angle calculation as performed by the software, on a metallic nanofilm. **B.** Contact angle evolution through time during this experiment. The last point for receding contact angle on Ti/Fe nanofilm is taken by removing the needle that perturbed the measurement.

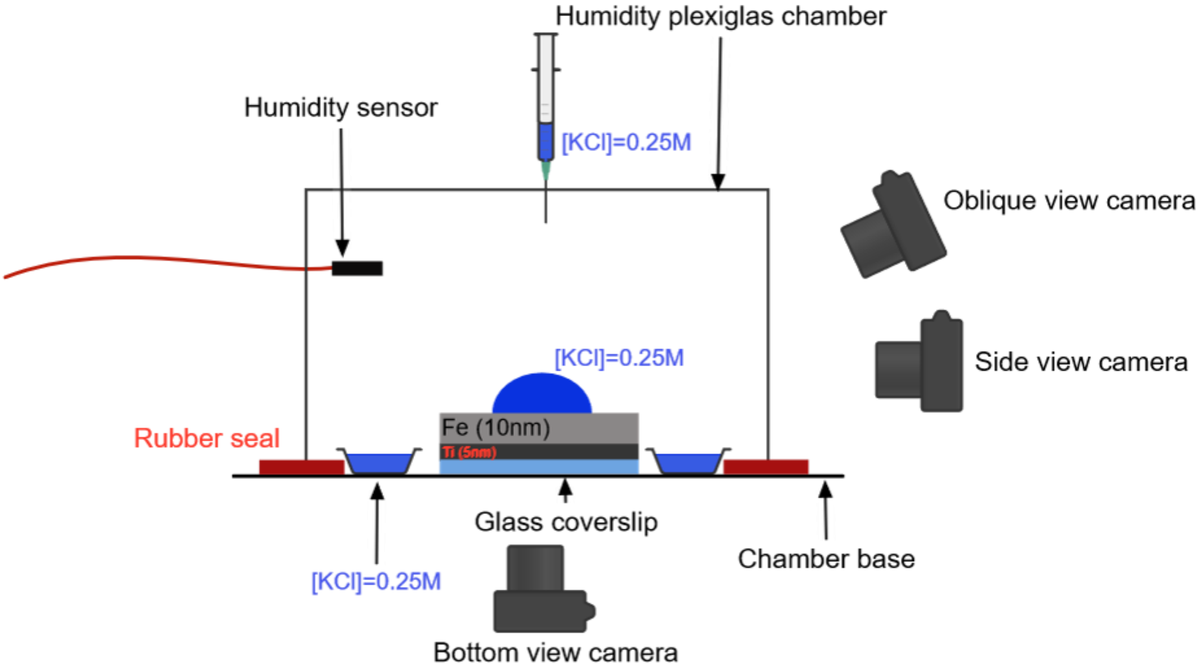

Figure S4 : Illustration of the setup for corrosion experiments created with chemix.org.

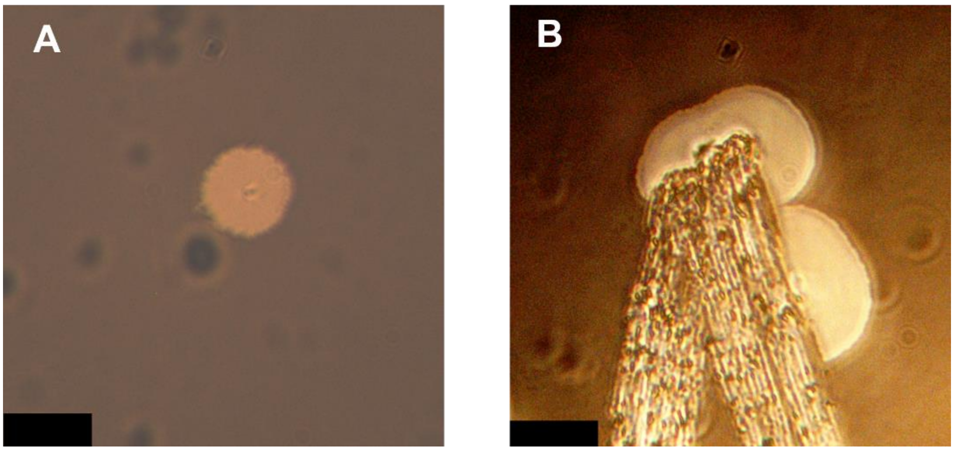

Figure S5 : Defects on nanofilms imaged under electrolyte using an optical microscope. Scale bars : 30 µm. Pictures were taken 1 minute after droplet deposition. **A.** Dust-induced defect starting a corrosion pit. **B**. Corrosion pits around a scratch manually performed.

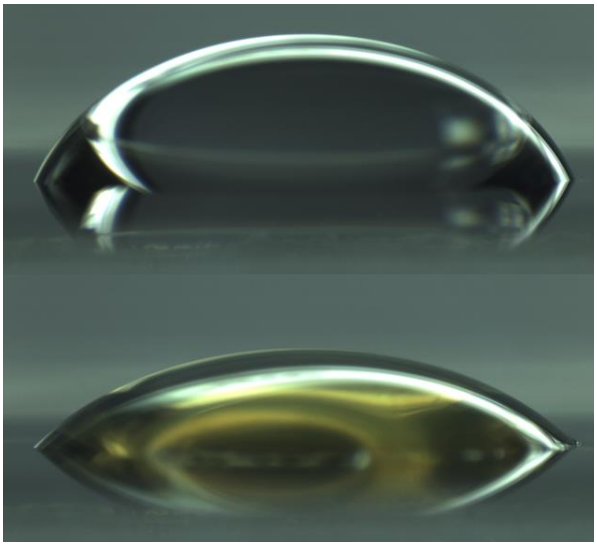

Figure S6 : Side view pictures from a preliminary experiment showing the yellow color appearing when corrosion begun. Here, images were chosen separated by 7 h for clarity reasons, but the phenomenon is clearly visible at short times (within 40 minutes). Top image = initial image recorded at t= 0h.

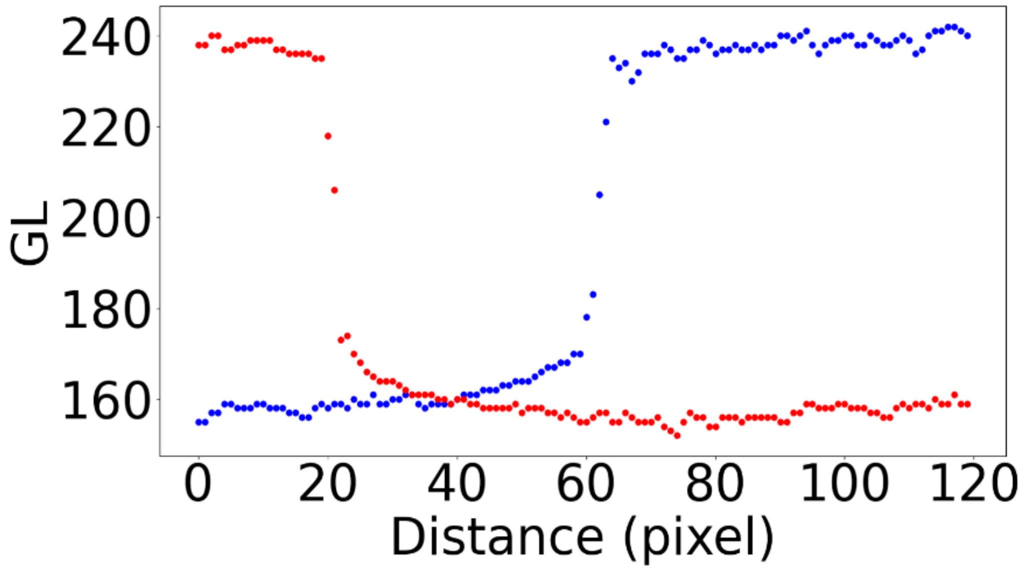

Figure S7 : Grey levels at sharp interfaces. Intensity profiles along a line, crossing a nanofilm edge (red) and a corrosion front (blue) for an Iron nanolayer. Note: 1 pixel is equivalent to 0.015 mm in the object plane.

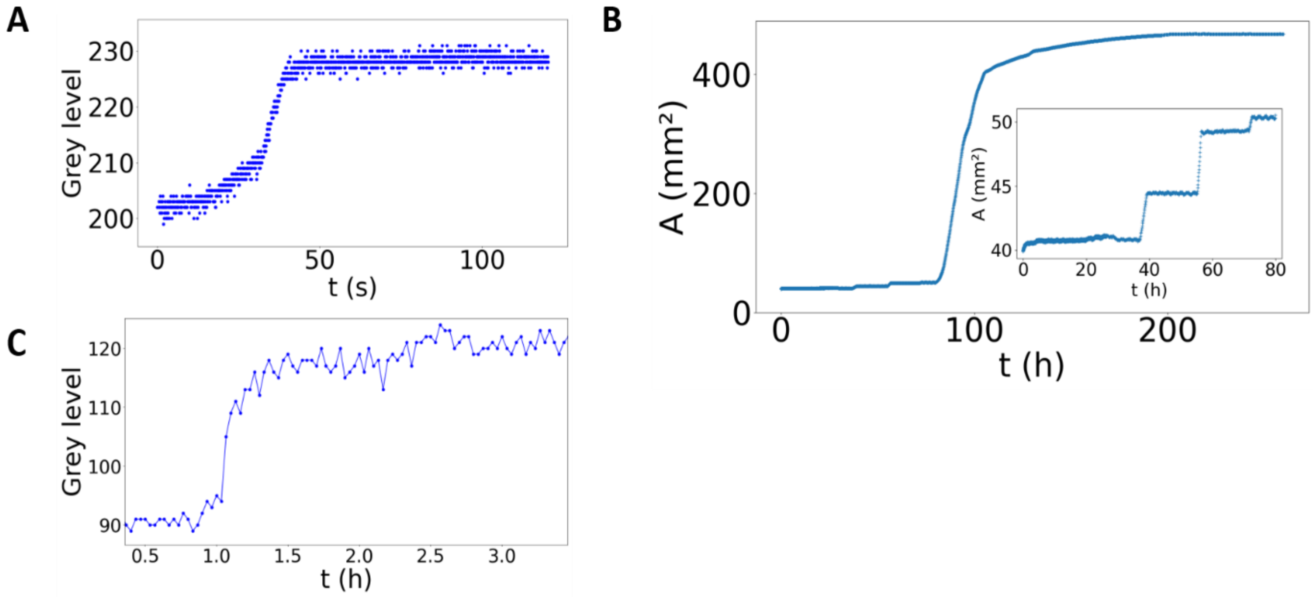

Figure S8: Corrosion evolution of a nanofilm. **A.** Temporal evolution of the grey level measured on one pixel for an iron nanofilm without titanium. **B.** Temporal evolution of the corroded area. **C.** Temporal evolution of the grey level measured on one pixel for an iron nanofilm with titanium.

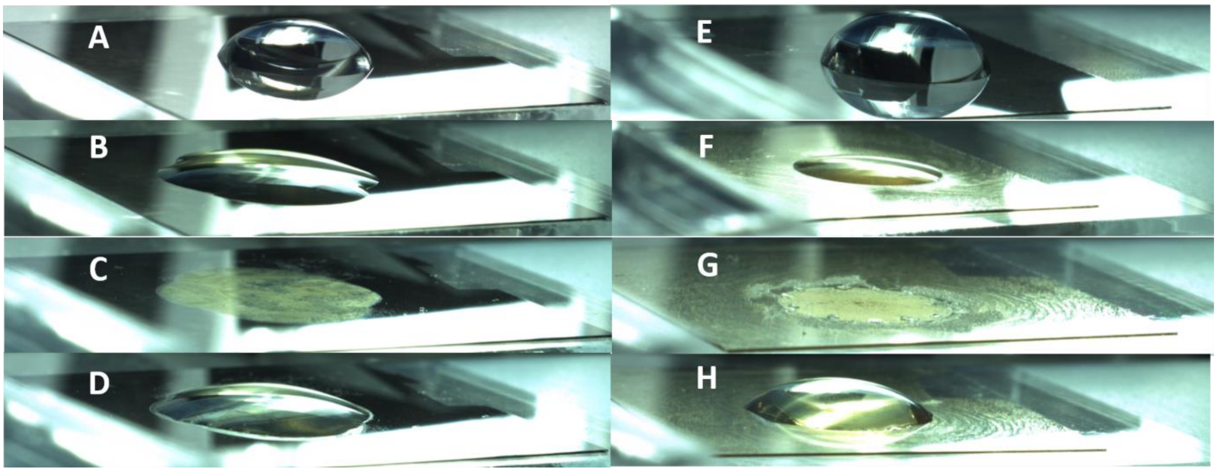

Figure S9: Wetting test (oblique views) just after corrosion with a KCl solution for a Fe/Ti nanofilm (**A**-**D**) and a Fe nanofilm without Ti (**E**-**F**). When the classic corrosion experiment was performed on a Fe/Ti nanofilm, with a droplet of volume *V= 70 µl*, at *t = 0 h* (**A**) and at *t = 5 h* (**B**). **C.** After drying with a Kimtech precision wipe. **D.** After a new droplet deposition (*[KCl] = 250 mM*, *V = 70 µl*). Note that the droplet has spread on almost the same surface as in **B**. We can notice the white line around the new triple line, which corresponds to a peripheral ring, initially corroded but not recovered by the new droplet. We could attribute this peripheral ring to the peripheral cathodic area depleted in oxide deposits and described in the classical corrosion process. When the experiment was performed on a Fe nanofilm without Ti, with a droplet of volume *V = 70 µl*, at *t = 0 h* (**E**) and at *t = 11 days* (**F**). **G.** After complete drying of the droplet, in contact with the atmosphere. **H.** Just after a new droplet deposition (*[KCl] = 250 mM*, *V = 70 µl*). We observe a greater contact angle in **H** than in **D**, suggesting that the hydrophilicity of the surface changed differently when corrosion proceeded on the titanium layer or on the glass. Comparison between **D** and **H** also indicates that the deposits on the titanium layer rendered the surface more hydrophilic than on glass surface. Note that the ring of precipitated salt has been cleaned with ultrapure water before droplet deposition, between **G** and **H**.

Substrate	Advancing contact angle (°)	Error (°)	Receding contact angle (°)	Error (°)	Static contact angle (°)	Error (°)
Ti 5nm	82	5	52	5	72	5
Ti 5nm +Fe 10 nm	69	5	25	10	76	10
Fe 10 nm	80	10	30	5	72	5

Table S1 : Average contact angles. Errors are estimated according to the large dispersion during the experiment.

Movie S1: Time lapse movie showing the corrosion experiment on a Fe/Ti (10nm/5nm) nanofilm. Different pictures correspond to oblique view (upper left picture), side view (lower left picture) and bottom view (right side picture). Scale bars indicate 2mm and contrast was enhanced numerically.

Movie S2: Time lapse movie showing the corrosion experiment on a Fe (10 nm) nanofilm. Different pictures correspond to oblique view (upper left picture), side view (lower left picture) and bottom view (right side picture). Scale bars indicate 2 mm and contrast was enhanced numerically.

Movie S3: Time lapse movie showing the corrosion experiment on a thick iron sheet (100 µm). Different pictures correspond to oblique view (upper picture) and side view (lower picture). The scale bar indicates 2 mm and contrast was enhanced numerically.
